# The Wound-Healing Effect of Mango Peel Extract on Incision Wounds in a Murine Model

**DOI:** 10.3390/molecules27010259

**Published:** 2022-01-01

**Authors:** Lesslie Espinosa-Espinosa, Leticia Garduño-Siciliano, Mario Rodriguez-Canales, Luis Barbo Hernandez-Portilla, Maria Margarita Canales-Martinez, Marco Aurelio Rodriguez-Monroy

**Affiliations:** 1 Laboratorio de Investigación Biomédica de Productos Naturales, Facultad de Estudios Superiores Iztacala, UNAM, Tlalnepantla C.P. 54090, Mexico; biol.lespinosa@gmail.com (L.E.-E.); mario.rodcan09@gmail.com (M.R.-C.); 2 Laboratorio de Toxicología de Productos Naturales, Escuela Nacional de Ciencias Biológicas, IPN, U. Prof. Adolfo López Mateos, Mexico City C.P. 07738, Mexico; lsicilia@hotmail.com; 3 Laboratorio de Farmacognosia, UBIPRO Facultad de Estudios Superiores Iztacala UNAM, Tlalnepantla C.P. 54090, Mexico; dra.margaritacanales@gmail.com; 4 Laboratorio Nacional en Salud, Facultad de Estudios Superiores Iztacala UNAM, Tlalnepantla C.P. 54090, Mexico; lbarbo@unam.mx

**Keywords:** *Mangifera indica* peel, wound healing, antibacterial, antioxidant

## Abstract

*Mangifera indica* can generate up to 60% of polluting by-products, including peels. However, it has been shown that flavonoids and mangiferin are mainly responsible for the antioxidant, anti-inflammatory, and antibacterial activities closely related to the wound-healing process. The chemical composition of MEMI (methanolic extract of *M. indica*) was analyzed by HPLC-DAD, as well as concentrations of total phenol (TPC) and flavonoids (TFC) and antioxidant activity (SA_50_). Wound-healing efficacy was determined by measurements of wound contraction, histological analysis, and tensiometric method; moreover, anti-inflammatory, antibacterial, and acute dermal toxicity (OECD 402) were also evaluated. Phenol, resorcinol, conjugated resorcinol, and mangiferin were detected. TPC, TFC, and SA_50_ were 136 mg GAE/g, 101.66 mg QE/g, and 36.33 µg/mL, respectively. Tensile strength and wound contraction closure did not show significant differences between MEMI and dexpanthenol groups. Histological analysis (after 14 days) shows a similar architecture between MEMI treatment and normal skin. MEMI exhibits a reduction in edema. *Staphylococcus epidermidis* had an MIC of 2 mg/mL, while *Staphylococcus aureus, Pseudomonas aeruginosa*, *and Escherichia coli* reached 4 mg/mL. The MEMI showed no signs of toxicity. Therefore, this study demonstrates multiple targets that flavonoids and mangiferin of MEMI may present during the healing process.

## 1. Introduction

In the last 25 years, the horticultural sector has grown both in area and production in order to satisfy the global demand for food [1]. According to the Food and Agriculture Organization of the United Nations (FAO), around 1300 million tons of food is wasted per year around the world, with fruits being the greatest loss (500 million tons)due to the industrial processes they are subjected to [2]. Mango (*Mangifera indica*) is one of the most important tropical fruits in the world due to its nutritional, economic, medicinal, and phytochemical properties, which leads to it being widely produced in various countries. Mexico ranks fifth worldwide in mango production and is marketed in 27 international destinations. Its main industrialization is that of pulp juices and nectars; however, 35% to 60% of the by-products are discarded after this manufacturing process, mainly peels and seeds [3].

On the other hand, ethnobotanical and pharmacological studies have mentioned the use of various parts of the mango tree (roots, barks, leaves, fruits, and seeds) to alleviate different gastrointestinal ailments, respiratory diseases, urinary infections, insect bites, and wounds [4]. These healing properties are attributed to their secondary metabolites, mainly polyphenolic compounds (PCs); the most reported PCs being flavonoids and mangiferin (glycosylated xanthone), and the peel presents higher amounts of these metabolites compared to pulp. These phytochemicals are characterized by having the ability to scavenge free radicals, activate endothelial cell migration, disrupt the cell membrane of microorganisms, and inhibit inflammatory and pain pathways [5,6].

Wounds are damages that affect the integrity and continuity of the epithelium, originating from different causes. They can be classified in different ways. One way is by the recovery time (acute or chronic). Acute wounds heal in less than 30 days, and chronic wounds heal in more than 30 days [7]. Among the acute wounds are the superficial ones, in which the damage occurs in the epidermis and the superficial part of the dermis, sometimes affecting the subcutaneous tissue. Wound healing is a complex physiological process aimed at repairing wounds. This process has four elementary phases that interact in an orderly manner: coagulation, inflammation, proliferation, and remodeling, provided that the appropriate conditions are in place to take place. However, the healing of acute wounds is usually not treated urgently, but it can be compromised by different factors: diabetes, stress, nutrition, chemotherapies, and infection, which can interfere with one or more phases of healing, causing serious complications and requiring specialized and very expensive medical attention [8]. In 2015, members of the Consejo consultivo de la Asociación Mexicana para el Cuidado Integral y Cicatrización de Heridas A.C. (AMCICHAC) indicated that the approximate cost to heal a (chronic) wound is USD 140 per week and that most of them could be preventable at the time they were acute injuries.

Recently, the clinical field has begun to focus on the treatment and care of these wounds as they have become quite a serious public health problem. The therapies that are implemented in superficial wounds are conventional that consist of managing the bacterial load with asepsis and debridement, delimiting the wound with patches and bandages, changing the bandage at least three times per day and complementing with the use of ointments that present a single chemical compound that acts on one or a maximum of two phases of the healing process, which becomes a painful and expensive therapy in the long term [9]. Therefore, it is necessary to carry out research and find new alternatives to their treatment; among that, natural products have generated great interest in research, and researchers are considering a secondary manufacturing process to extract bioactive compounds from natural products as a source of phytochemicals [10]. For these reasons, the aim of this study is to identify the chemical composition of the mango peel extract and evaluate the antioxidant, antibacterial, anti-inflammatory, and healing effects in incision wounds in a murine model.

## 2. Materials and Methods

### 2.1. Chemicals and Reagents

Aluminum chloride (AlCl_3_), 2,2-diphenyl-1-picrylhydrazyl (DPPH°), Folin–Ciocalteu’s reagent, quercetin, sodium carbonate, methanol, and authentic mangiferin standard were purchased from Sigma-Aldrich (St. Louis, MO, USA). 2,3 5-Triphenyl-2H-Tetrazolium chloride (TTC) CAS 298-96-4 was obtained from Solarbio Life Science. All chemicals and reagents used were of analytical grade. The bacterial strains were grown in nutrient Müeller Hinton broth (Bioxon 260–1, Estado de Mexico, Mexico). For the disc diffusion technique, Müeller Hinton agar (Bioxon, Edo. de Mexico, Mexico) was used.

### 2.2. Samples Collection

The fresh fruit *M. indica* L. variety “Ataulfo” were obtained from a local organic garden in San Jerónimo of Juárez, in the municipality Benito Juárez, Guerrero state. The mango peels were washed with pure water and then carefully separated and removed from the mango pulp with a knife. Then, the mango peels were dried at room temperature. The dried mango peels were sliced into smaller pieces.

### 2.3. Preparation of Methanolic Extract from Mango Peels

The methanolic extract of *M. indica* (MEMI) was obtained by maceration [11] fdd. One kilogram of dried mango peels is macerated with methanol for five days in a conical flask. The extract was filtered and then distilled under reduced pressure on a rotary evaporator. Subsequently, the distillate was evaporated in a fume hood until it was dry (381.591 g). Subsequently, the yield percentage was calculated based on the initial weight of the dry plant material and provided a yield percentage of 38.12% (*w*/*w*). For in vivo models, MEMI was prepared in a gel-based formulation (1% Carbomer Gel 940 in deionized water as a vehicle) at a concentration of 10% (*w*/*w*). One gram of the crude extract of MEMI was separated into 10 mL of the vehicle by magnetic stirring. Once the ointment had an excellent physical appearance (homogeneous and transparent), it was stored under refrigeration until used in the experiments.

### 2.4. High-Performance Liquid Chromatography (HPLC-DAD) Analysis

The MEMI was analyzed by high-performance liquid chromatography (HPLC) on a Hewlett Packard high-resolution liquid chromatograph, the Agilent Technologies series model 1100, equipped with a diode array detector (DAD) and Agilent ChemStation A0903 software for the LC system. Thirty microliters of MEMI were injected into the system. The extract was prepared at a concentration of 3 mg/mL. A 250 mm long Allosphere ODS-1 column with an internal diameter of 4.6 mm and a particle size of 5 μm was used as the stationary phase at a column temperature of 25 °C. The mobile phase consisted of a concentration gradient of MeOH-acetonitrile-H_2_O with the following conditions: 0–3 min (0:10:90), 3–5 min (25:10:65), 5–15 min (40:10:50), and 15–30 min (90:10:0). Full scanning of 220–400 nm was performed in an execution time of 30 min with a constant flow rate of 1 mL/min and a detector array of diodes with the detector set at 256 nm. The constituents were identified based on a comparison of the retention time and UV spectrum with those of the standards (mangiferin and quercetin 3-β-D-glucoside).

### 2.5. Liquid Chromatography-Mass Spectrometry (HPLC-MS) Analysis

HPLC-MS analysis was performed using an Agilent 1200 Infinity LC coupled to an Agilent 6230 TOF mass spectrometer with an Agilent Dual ESI Source (ESI SG14289023) and MassHunter Workstation Software, version B.05.01, Build 5.01.5125.3, operating in negative ionization mode. The capillary voltage was 4500 V; the dry gas temperature was 300 °C; nitrogen was used as the dry gas at a flow rate of 7 L/min; the nebulizer pressure was 60 psig; the fragmentor was 200 V; the MS range was 50–1500 *m*/*z*; and the MS acquisition rate was 1 spectrum/s. The chromatographic separation was accomplished using an HPLC system (Infinity Series 1200, Agilent Technologies, Waldbronn, Germany) equipped with a Kinetex 2.6 u, C1800A column (150 × 2.1 mm) (Phenomenex, SA, Torrance, CA, USA). The column temperature was maintained at 25 °C. The following gradient program was used along with a mobile phase consisting of water: acetonitrile (90:10) with 1.0% formic acid (solvent A) and methanol:acetonitrile (90:10) with 1.0% formic acid (solvent B). These initial conditions were 3 min in an isocratic elution composed of 100% solvent A followed by 3–5 min: 65% A-35% B; 5–15 min: 50% A-50% B; 15–30 min: 100% B; and 40 min: 100% B, v/v. The flow rate was 0.15 mL/min, and the injection volume was 6 μL (0.1 mg/mL) for the mangiferin standard and 10 μL (0.3 mg/mL) for methanolic extract.

### 2.6. Determination of Total Phenolic Content

The total phenolic content in MEMI was based on a colorimetric oxide reduction reaction of the Folin–Ciocalteu reagent [12]. A calibration curve was prepared with serial concentrations of acid gallic (0.00625, 0.0125, 0.0250, 0.05, 0.1, 0.2 mg/mL). The absorbance was measured at 760 nm using a UV–Vis spectrophotometer (DU 640 Spectrophotometer, Beckman, Brea). The stock solution (0.2 mg/mL) of MEMI was prepared with 500 μL of Folin–Ciocalteu reagent and allowed to act for 5 min, then a solution of Na_2_CO_3_ was added. Finally, the resulting mixture was allowed to stand for two hours at room temperature. The sample was tested by triplicate. The concentration against absorbance was graphed to obtain a standard curve of gallic acid. By linear regression analysis, the absorbance of the test sample was interpolated. The results were reported as gallic acid equivalents (mg GAE/g extract).

### 2.7. Determination of Total Flavonoids Content

The total flavonoid content present was determined by the Dowd method [13]. Using a solution of quercetin dissolved in MeOH HPLC grade (1:1), aliquots of this solution for the preparation of the standard curve were taken with the characteristics of 1–100 mg/mL, and 1 mL of aluminum chloride (AlCl_3_) 2% MeOH HPLC grade was added. The MEMI extract was diluted in MeOH HPLC grade in the same concentration as stock of quercetin and 2% AlCl_3_. One milliliter of the extract solution and one milliliter MeOH without AlCl_3_ was the blank sample. In an ELISA plate, aliquots by triplicate of the standard curve of the sample and the blank were placed. After 10 min at room temperature in the dark, the absorbance was measured at 450 nm in a Bio-Tek EL800 plate reader (Bio-Tek), and the total flavonoid content was expressed as milligrams of quercetin equivalent per gram of extract (mg of QE/g of extract).

### 2.8. Free Radical Scavenging Activity

The half antioxidant capacity (SA_50_) of MEMI samples was 1–100 ppm, and quercetin as control positive was determinate [14]. We used the radical DPPH° (2,2difenil-1-picrilhidracil) and blank solution (MeOH HPLC grade). Aliquots of 50 μL of the tested sample were placed in an ELISA plate and 150 μL freshly prepared DPPH° solution in methanol and shacked. Immediately, it was incubated for 30 min at 37 °C in darkness. The absorbance was read at 540 nm using a Bio-Tek EL800 plate reader (Bio-Tek). DPPH° radical-scavenging capacity was calculated as:

(1) $$\%\;\mathrm{reduction}=\frac{\left[\left(\mathrm{absorbance}\;\mathrm{of}\;\mathrm{control}-\mathrm{absorbance}\;\mathrm{of}\;\mathrm{sample}\right)\right]}{\mathrm{absorbance}\;\mathrm{of}\;\mathrm{sample}\;}\times 100$$

According to [15], the antioxidant activity index of MEMI was determined as poor, moderate, strong, or very strong (AAI = <0.5, between 0.5–1.0, between 1.0–2.0 or >2.0, respectively), with the next formula:

(2) $$\mathrm{AAI}=\frac{\mathrm{final}\;\mathrm{concentration}\;\mathrm{of}\;\mathrm{DPPH}\;\left(\mu g/\mathrm{mL}\right)\;}{{\;\mathrm{SA}}_{50}\;\left(\mu g/\mathrm{mL}\right)\;}\;$$

where: AAI = Antioxidant activity index; SA_50_ = Scavenging Antioxidant medium.

### 2.9. Experimental Animals

Healthy adult male CD1 mice and healthy female Wistar rats were used. The animals were left for 5 days in the environmental conditions for acclimatization. They were kept on a standard diet of pellets and water ad libitum throughout the experiment. The animals were kept at a constant temperature (22 ± 2 °C) and 50 ± 5% relative humidity on a 12-h light-dark cycle. The animals were treated according to the guidelines of the Federal Regulations for Animal Experimentation and Care (NOM-062-ZOO-1999, Ministry of Agriculture, Mexico); this work was approved by the Comité de Ética e Investigación de la Escuela Nacional de Ciencia Biológicas del Intituto Politécnico Nacional (CEI-ENCB ZOO-004-2020), and by the Comité de Ética de la Facultad de Estudios Superiores Iztacala, Universidad Nacional Autónoma de México (CE/FESI/052019/1295).

### 2.10. Study of Wound Healing Efficacy

#### Measurement of Wound Contraction

From this study, 18 mice were utilized, which were divided into three groups containing six animals each and treated, respectively, with 10% of MEMI ointment, dexpanthenol 5% (a commercial product containing a 5% dexpanthenol water-in-oil emulsion was used, Bepanthen^®^ cream, Bayer, Germany), or vehicle (surgical gel 1% Carbomer Gel 940 in deionized water). The hair on the back of all mice was shaved using a shaver machine and then was depilated with a body hair removal cream 24 h before the study began. The animals were anesthetized with 5% isoflurane via inhalation. Afterward, a longitudinal wound of 1 cm was made with a scalpel number three, considering only the dermis and epidermis layers. The tests were applied via topic each 12 h for 14 days [16]. Instantly, each mouse was collocated on separated boxes until all the treatment was absorbed. On day 15, the animals were sacrificed using a CO_2_ chamber.

The wound-healing progress was measured every day with ImageJ2 software. The percentage of wound contraction was evaluated using the initial size of the wound (1 cm) as 100%, as shown in the following way:

(3) $$\%\;\mathrm{wound}\;\mathrm{contraction}=\frac{\mathrm{initial}\;\mathrm{wound}\;\mathrm{size}-\mathrm{specific}\;\mathrm{day}\;\mathrm{wound}\;\mathrm{size}}{\mathrm{initial}\;\mathrm{wound}\;\mathrm{size}}\times 100$$

### 2.11. Histological Analysis

Wound tissues of the different treatments were obtained and fixed on 4% formalin solution. Twenty-four hours after, they were dehydrated in alcohols of increasing concentration until reaching absolute alcohol. Afterwards, the tissue was passed through intermediate solvents, such as xylene, and the samples were preserved in paraffin wax. It was obtained 5 μm thick sections and stained with hematoxylin–eosin (H&E) to observe under light microscopy, and the wound closure length was analyzed with ImageJ2 software.

### 2.12. Tensiometric Method

A total of 24 CD-1 mice were distributed in four groups with six animals in each. The groups were treated as follows: the first group was normal skin; the second group had dexpanthenol (5%) treatment as a positive control; the third group had MEMI (10%) ointment treatment; and the fourth group had the vehicle (surgical gel) treatment. Only the animals in the first group were shaved, and none were wounded. The experimental time, the wound, and the sacrifice were conducted as the methodology before.

Immediately after the sacrifice, the wound-healing contraction was measured using the technique of water flow. Subsequently, the percentage of healing efficacy was determined using the following equation:

(4) $$\%\;\mathrm{wound}\;\mathrm{healing}\;\mathrm{efficacy}=\frac{A0\times An\;}{A0}\times 100$$

where *A*0 refers to the initial wound size and *An* to the wound size on a specific day.

### 2.13. Assay of TPA-Induced Inflammation in Mice

12-O-tetradecanoylphorbol-13-acetate (TPA)-induced skin inflammation causes increased ear thickness and skin water content in mice [17]. Twenty-four mice were used for this test and fasted for four hours before the test. Later, they were randomized into four groups with six animals in each (*n* = 6). A volume of 10 μL was administered on the internal and external surfaces of the right ear to induce skin inflammation. As a reference drug, 0.5 mg of MEMI 10%, diclofenac (0.116 mg) was administered, and surgical gel was administered as a vehicle (0.5 mg). Both were applied topically 30 min before treatment with TPA. The left ears were considered the control group.

Once the different treatments were applied, they were allowed to act for four hours. Subsequently, the animals were sacrificed by cervical dislocation, and with the help of a biopsy punch, the left and right ears were cut, respectively. The samples were immediately prepared for histological analysis, microscopic observation, and inflammatory thickness measurement with ImageJ2 software. The anti-inflammatory percentage was obtained by measuring the thickness of the ear of the TPA group (a), which was considered 100% inflammation, the control group (b), and the treatment groups with TPA (c). The following values were subsequently calculated [18]:

Edema A induced by TPA alone (*a* − *b*)

Edema B induced by TPA plus treatment (*c* − *b*)

Anti-inflammatory percentage (%) = [(Edema A−Edema B)/Edema A]∗100

### 2.14. Evaluation of Antibacterial Activity In Vitro

#### 2.14.1. Evaluation by Kirby–Baüer Agar Diffusion Method

Four bacterial strains with relevant clinical importance in wound healing were utilized: *Staphylococcus aureus*, *Staphylococcus epidermidis* (both isolated from a clinical case)*, Pseudomona aeruginosa* (donated by the CINVESTAV), and *Escherichia coli* (isolated from a clinical case). The bacterial strains were grown in 10 mL of nutrient broth Müller–Hinton at 37 °C for 24 h, which has been adjusted to 0.5 McFarland standard. Sterile discs of 5 mm diameter made of Whatman paper of No. 5 were impregned with 2 mg of MEMI dissolved in 1 mL of MeOH. Chloramphenicol was included as a positive control (25 μg/disc), and negative control was sterile disc impregned with MeOH (10 μL). All tests were carried out for five sample replications, and the bacterial inhibition was evaluated by measuring the diameter of the clear zone around the disc [19].

#### 2.14.2. Minimal Inhibitory Concentration and Minimal Bactericide Concentration Determination

The minimal inhibitory concentration (MIC) and minimal bactericide concentration (MBC) were determinate by the broth dilution method [19]. The concentrations evaluated were: 8, 4, 2, 1, 0.5, 0.25, and 0.125 mg/mL. In a 96-well plate, 50 μL of Müller–Hinton broth was added. At that time, 100 μL of the stock solution was placed, and dilutions were made. Fifty milliliters of bacterial cultures were inoculated with a concentration of 1 × 10^5^ UFC/mL. The plate was incubated for 24 h at 37 °C. Finally, 50 μL of a 0.08% solution of TTC (tetrazolium chloride) was added. The lower and the lowest concentrations that inhibit the bacteria growth were considered MIC and MBC, respectively.

### 2.15. Acute Dermal Toxicity Test

The acute skin toxicology study was conducted in accordance with the OECD guideline 402 adopted in October 2015 [20]. A total of nine Wistar female rats weighing 200–220 g were randomly selected with three animals for each group. One animal was distributed per box and placed under standard conditions housed for a week for acclimation. A MEMI 10% ointment was prepared with surgical gel as a vehicle. The limited test was 2000 mg/kg, while the vehicle group received 500 mg/kg of surgical gel and the control group received no treatment. The dorsal area (10%) of each animal was shaved. After 24 h, all doses were only applied locally once on the first experimental day and covered with a cotton dressing (which was impregnated with 2 g of surgical gel to saturate its absorption level). For 14 days, the rats were monitored and weighed every third day. It was determined if the treatments cause unfavorable reactions, such as changes in physical appearance, behavior patterns, injuries, pain, signs of illness, and death. On day 15, the animals were sacrificed by cervical dislocation.

### 2.16. Statistical Analysis

Antibacterial activity and tensiometric method results were expressed as the mean ± standard deviation or error of the mean (S.D. or S.E.M.), respectively. Analysis of the data was conducted using the one-way analysis of variance (ANOVA) with a Tukey–Kramer multiple comparison post hoc test with a significance value of (*p* < 0.05). The wound contraction measurement results were expressed as the mean ± S.D. or S.E.M. The analysis of the data was conducted using a significance value of (*p* < 0.05). All the analyses were carried out on GraphPad Prism 7 software.

## 3. Results

### 3.1. Chemical Characterization

**Figure 1:**
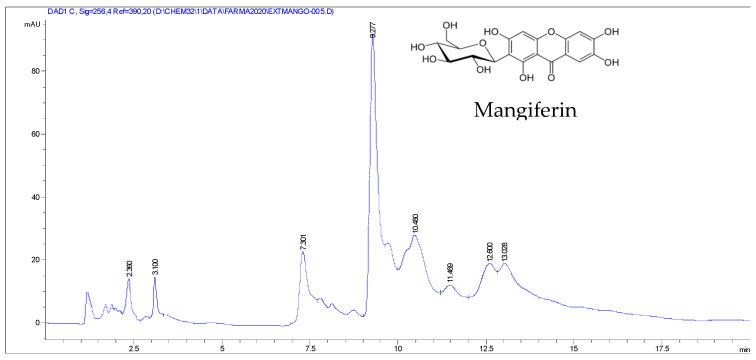
HPLC-DAD chromatogram of the methanolic extract of *M. indica* obtained with a wavelength of 256 nm.

**Figure 2:**
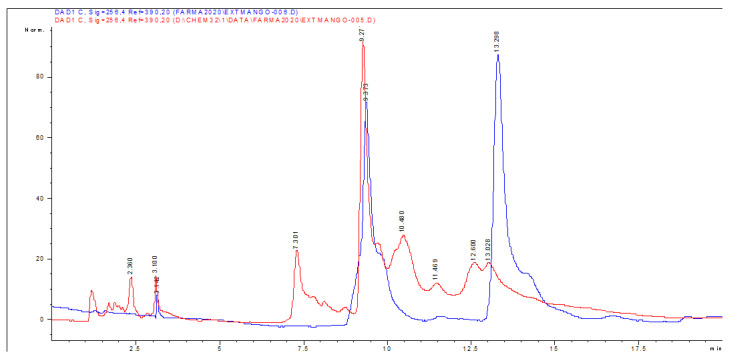
HPLC-DAD chromatogram of the methanolic extract of *M. indica* (red line) and of the standard glycosylated mangiferin and quercetin 3-β-D-glucoside obtained with a wavelength of 256 nm (blue line).

**Figure 3:**
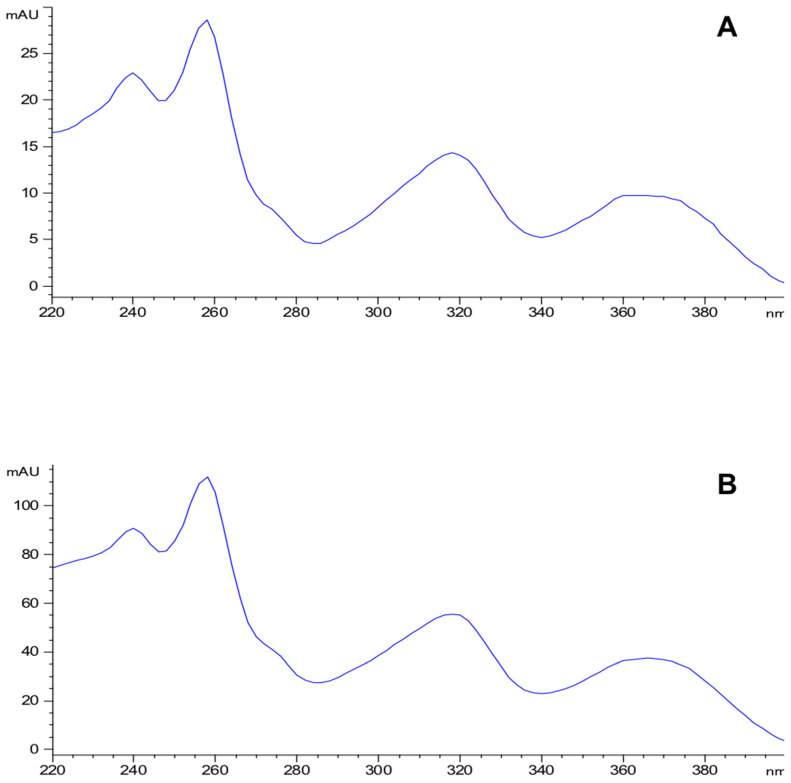
Ultraviolet light spectra of the mangiferin standard (**A**) and the compound detected in the methanolic extract of the exocarp of *M. indica* (**B**).

**Figure 4:**
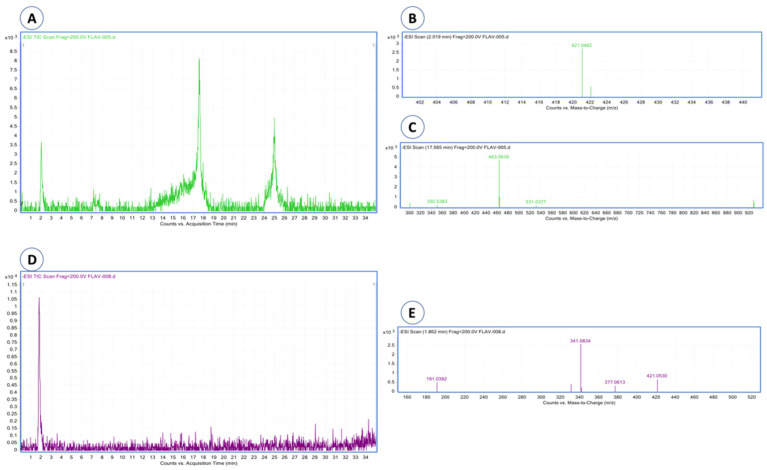
HPLC-MS chromatograms and mass spectra of mangiferin and quercetin 3-β-D-glucoside were detected in mango extract. (**A**) Chromatogram of the mangiferin and quercetin standards; (**B**) mass spectrum of mangiferin; (**C**) mass spectrum of quercetin; (**D**) chromatogram of the mango extract, where mangiferin is detected; (**E**) mass spectrum of mangiferin detected in mango extract.

Eight compounds were detected at 256 nm (Figure 1) in MEMI, of which mangiferin was identified (Rt = 9373 min). Based on the area under the curve, the percentage of mangiferin area per gram was calculated (2.26%) (Figure 2). To verify these aspects, the ultraviolet light spectra of both were obtained observing that they also coincide in the maximum absorption peaks (240, 258, 318, and 366 nm) and chromatogram structure (Figure 3). Mangiferin was also identified by HPLC-MS (Table 1, Figure 4).

Moreover, phenol (Rt = 2.36 min), resorcinol (Rt = 3.1 and 7.301 min), and resorcinol conjugate (Rt = 10.48, 11.469, 12.60, and 13.028 min) also were identified.

### 3.2. Quantify of Polyphenols, Flavonoids Content, and Antioxidant Capacity

The amount of the total polyphenols and flavonoids in the MEMI are shown in Table 2. The total phenolic content displayed was 136 mg GAE/g, and the total flavonoid content was 101.66 mg QE/g. These compounds are related to a strong antioxidant capacity. Therefore, by the DPPH° method, the medium antioxidant capacity (SA_50_) of MEMI was calculated, which was 36.33 μg/mL. In accordance with the antioxidant activity index [15], the MEMI has a very strong antioxidant capacity, which is AAI = 3.72.

### 3.3. Measurement of Wound Contraction

**Figure 5:**
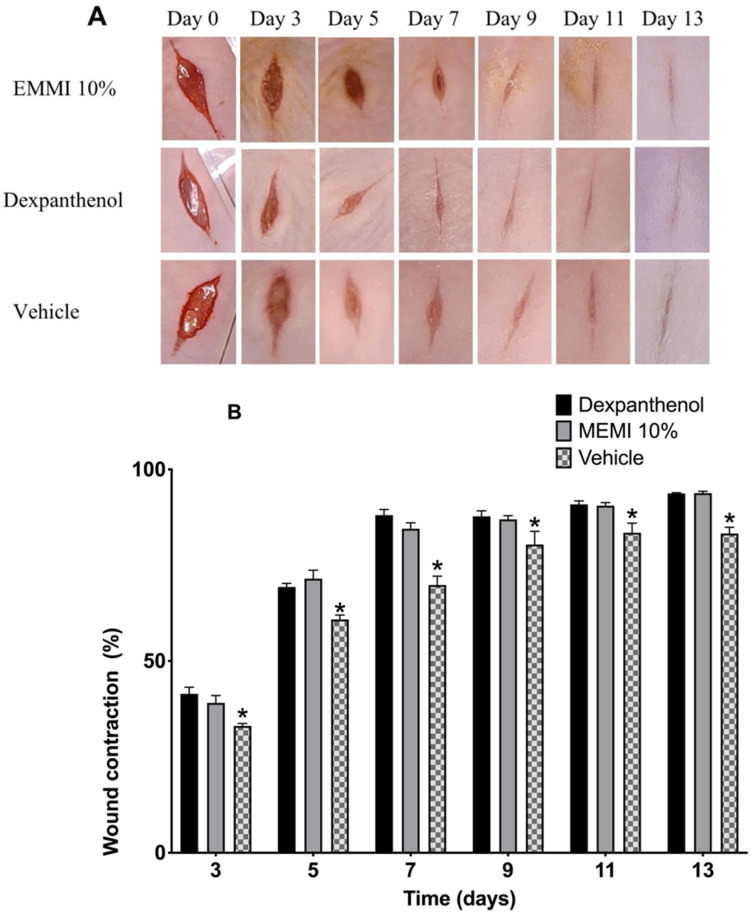
Effects of MEMI 10% on wound’s contraction. (**A**) Representative photographs of the wounds of mice treated with MEMI 10%, dexpanthenol (control), and surgical gel as a vehicle. The photographs of the wounds at 0–13 days are representative of six mice in each group. (**B**) Percentage reduction in wound size in control, MEMI 10%, and vehicle groups. All values are expressed as the percentage of wound contraction ± SEM. Two-way ANOVA support Tukey multiple comparisons test analysis and showed that both dexpanthenol and MEMI 10% treatments have similar wound closure. * *p* < 0.05 compared to dexpanthenol and MEMI 10% groups.

The wounds of the dexpanthenol group showed a slightly more aesthetic closure than the MEMI 10% group. However, in both groups, granulation tissue was observed starting on the third day after the lesion was performed. On the eleventh day, the scar began to be defined (Figure 5A). The length of the wound closure showed no significant differences (*p* < 0.05) during the 14 days of experimentation. Both groups, dexpanthenol and MEMI 10%, exceeded 50% closure after the fifth day (Figure 5B). On the other hand, the vehicle group showed significant differences with respect to the dexpanthenol and MEMI 10% groups (Figure 5B).

### 3.4. Histological Analysis

**Figure 6:**
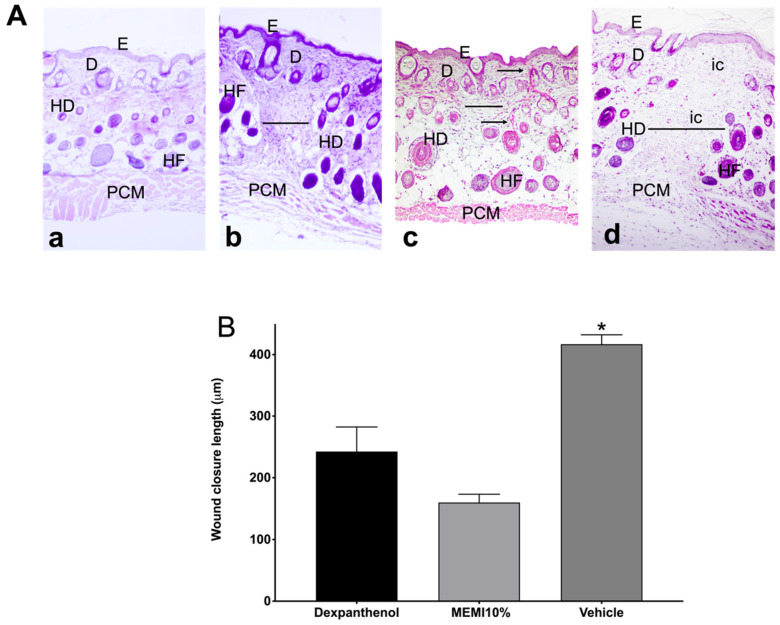
Histological analyses of MEMI. (**A**) Histological sections of skin wounds on mice. Control group (Dexpanthenol) showed a thicker epidermis than that of MEMI 10% group on wound´s contraction. Representative photographs of the wound’s architecture on the 14th day: normal skin (**a**); dexpanthenol group (**b**); MEMI 10% group (**c**); surgical gel vehicle (**d**); epidermis (E); dermis (D); hypodermis (HD); hair follicle (HF); panniculus carnosus muscle (PCM); inflammatory cells (ic); measurement wound (-); collagen fibers (←). Tissues were stained with H&E and visualized at 10× magnification. (**B**) Reduction in wound on 14th day of treatment. Results were expressed as the mean ± S.D. The analysis of the data was conducted using two-way ANOVA with a Tukey multiple comparison post hoc test. * *p* < 0.05 compared to dexpanthenol and MEMI 10% groups.

Tissue sections stained with H&E on day 14 were examined to determine the architecture of the skin layers, cell infiltration, wound closure length, and collagen fibers (Figure 6A). The skin without lesion shows three well-differentiated layers (epidermis, dermis, and hypodermis) and panniculus carnosus muscle, and all the layers are conformed orderly manner. In contrast, the MEMI and dexpanthenol groups showed the wound section. In this case, the MEMI group showed less wound closure length, better architecture in the epidermis and dermis layers than the dexpanthenol group, as well as the presence of collagen fibers. On the other hand, the dexpanthenol group evidenced infiltrating cells on the dermis layer different from the MEMI group. However, the wound measurements were not statistically significant between these groups (*p* < 0.05). In both treatment groups, the hypo-dermis layer is not completely formed compared to normal skin, which is observed in the three well-structured and definite layers. Conversely, the vehicle group showed greater wound length and a large amount of cellular infiltrate. Moreover, the vehicle group presented statistically significant differences with respect to the MEMI 10% treated group but not with respect to the dexpanthenol group (Figure 6B).

### 3.5. Tensiometric Method

**Figure 7:**
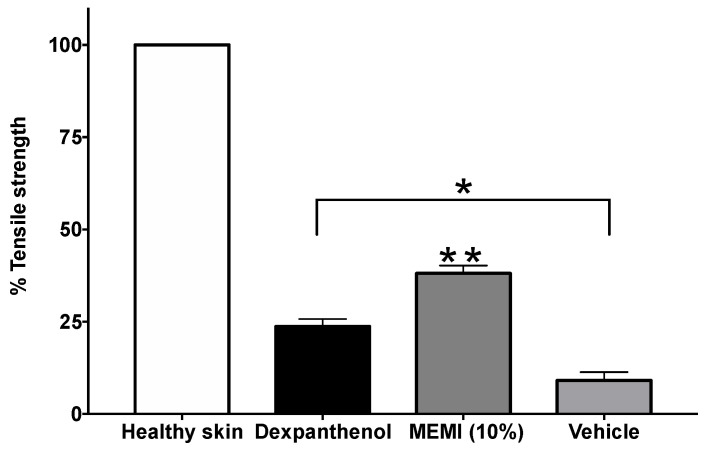
Percentages of tensile strength of MEMI 10%. The data are represented as means ± S.E.M (*n* = 6). * Significant difference compared to the skin healthy group (*p* < 0.05). ** Significant difference compared to the dexpanthenol and vehicle groups (*p* < 0.05).

Figure 7 shows the percentages of tensile strength in the dexpanthenol, 10% EMMI, and vehicle groups, there are significant differences with regards to the group with healthy skin. However, the 10% EMMI group was significantly increased by about 38.09% (*p* < 0.05) compared to the dexpanthenol (23.73%) and vehicle (9.08%) groups.

### 3.6. Anti-Inflammatory Activity by TPA Model

**Figure 8:**
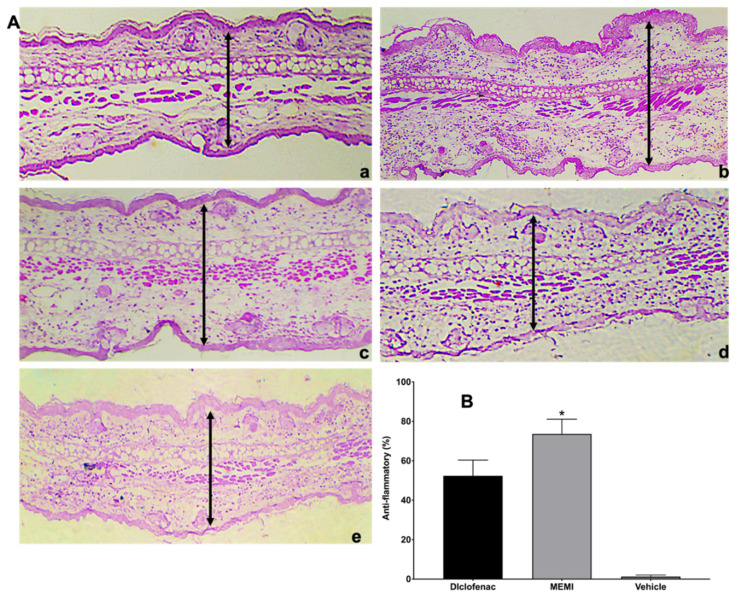
Histological analysis of MEMI. (**A**) Auricular edema histological sections (10×). (**a**) Control group (left ear), normal sample tissue. (**b**) Treated group with only TPA; it is noted that there is too much edema and leucocitary infiltrate. (**c**) Diclofenac-treated group presents moderate inflammatory infiltrate cells and edema. (**d**) group treated with MEMI 10%; moderate leucocitary infiltrate and less edema is noted. (**e**) Vehicle-treated group showed edema and moderate leucocitary infiltrate. The simples were dyed with H&E, and the black arrows indicate the measurement of the inflammatory thickness of the ears of the mice. (**B**) Anti-inflammatory percentage of the TPA model. There are no significant differences among MEMI and diclofenac groups. The results were expressed as the median ± S.D.M. Two-way analysis of variance was followed by the Tukey test. * *p* < 0.05 compared to the diclofenac and vehicle groups.

The histological analysis from MEMI showed interesting results for the different treatments. On the control group (left ear), a normal structure of the pinna was observed (Figure 8A(a)), while the group of TPA showed typical inflammatory signals (vasodilation, edema, and immune cell infiltrate) (Figure 8A(b)). On the other hand, the group treated with the MEMI extract evidenced a bit of edema, but leucocytes cell infiltrate was not less than the diclofenac group (Figure 8A(c,d)). The MEMI extract did not show significant differences with respect to the control positive group (Figure 8B). The vehicle group did not show significant differences with respect to the TPA treatment group and then did not present any anti-inflammatory effects in ear tissue (Figure 8A(b,e)).

### 3.7. Antibacterial Activity

Two Gram-positive strains bacterial (*S. aureus* and *S. epidermidis*) and two Gram-negative (*E. coli* and *P. aeruginosa*) strains with clinical importance were used because these compounds are often reported in wound infections. All the bacterial strains were sensitive to MEMI. *S. epidermidis* showed the biggest inhibition halos (13.8 ± 1.9) and the best MIC (2 mg/mL). Nevertheless, all strains reached the CMB with 8 mg/mL (Table 3).

### 3.8. Acute Dermal Toxicity

**Figure 9:**
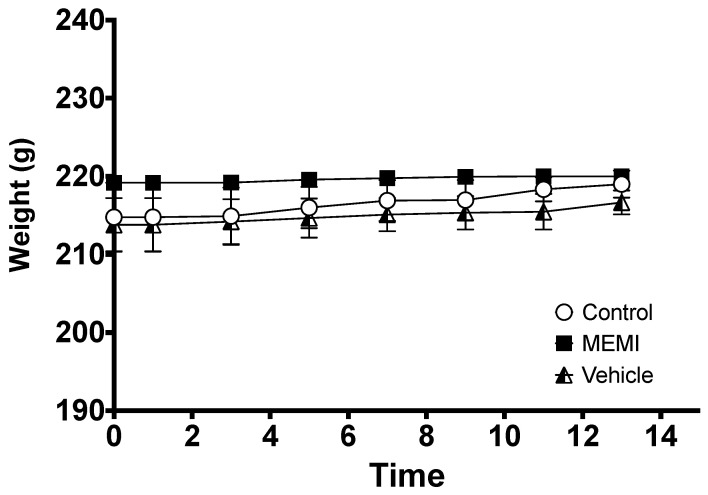
Effect of MEMI on the weights of female rats. All values are expressed as the mean ± SD (*p* < 0.05).

MEMI at a dose of 2000 mg/Kg is not toxic. After 24 h of applying MEMI, no sign of dermatological toxicity, such as changes in the skin, fur, eyes, mucous membranes, nor in the respiratory, circulatory, autonomic, and central nervous systems, somatomotor activity, and the pattern of behavior, tremors, convulsions, salivation, diarrhea, lethargy, sleep, and coma, were observed (Table 4) No weight loss (Figure 9) or mortality was observed, even after 14 days.

## 4. Discussion

Wound healing is one of the most complex and dynamic processes that mammals present at the biochemical and cellular levels. For years, natural products have been used to promote it because they are more accessible to the population and have lower costs compared to conventional therapies [21]. Mango fruit’s by-products are an alternative for the evaluation of biomedical properties because they present important chemical compounds that can benefit health [22].

Polyphenolic compounds are found mainly in mango peels, and together with glycosylated xanthones, triterpenoids, tannins, and derivatives of gallic acid, they generate very important biological properties related to healing [23]. The chemical profile obtained from MEMI by high-performance liquid chromatography showed different compounds: phenol, resorcinol, conjugates of resorcinol, and mangiferin. This coincides with studies of several cultivars of this by-product, in which it was shown that they are a mixture with abundant polyphenolic biosynthesis compounds [24], and their predominant compound is mangiferin [1]. Although different authors have pointed out that the chemical profile of mango peels is more complex, it is known that xanthones are characteristic of *M. indica* because they are present in all plant structures [25,26].

In this study, the results of TPC and TFC were 136 mg eAG/g and 101.66 mg eQ/g, corresponding to 13.6% and 10.1%, respectively. These values differed considerably with the extracts of the pulp of the same variety collected in the state of Guerrero with a TPC of 5.15 mg eAG/g and TFC of 4.07 mg eQ/g [23]. On the other hand, the peel extracts of var. “Irwin” showed a higher TFC than the pulp extracts, increasing up to 29 times more. Likewise, the peel extracts of vars. “Golden” and “Aust R2E2” presented 10.32 mg eAG/g and 10.98 mg eAG/g, respectively [25]. The peel is the first physical barrier that the fruit presents to protect the seed from any physical, chemical, or biological factor; therefore, it must synthesize a higher content of phenolic compounds compared to the pulp to provide that protection [25].

Polyphenols, specifically flavonoids, significantly contribute as antioxidant-reducing agents, hydrogen donors, and free radical inhibitors. The measurement of this capacity was determined by the DPPH° method, and MEMI had an SA_50_ of 36.33 ppm, presenting an AAI of 1.10; therefore, it is classified as having strong antioxidant activity [15]. Different extracts of the mango peel have reported varied data of the SA_50_: var. “Irwin”, 31.25 ppm in ethanolic extracts, var. “Tommy Atkins”, 188 ppm lyophilizates, var. “Sidhura”, 65.35 ppm in aqueous extracts [27,28,29], respectively.

In addition to the above, it is well known that crude extracts have more and better biological properties than pure compounds due to the complex mixture of metabolites that are products of different biosynthetic origins. In the study by [30], the lyophilizates of *M. indica* against the standard compound (mangiferin) demonstrated a higher antioxidant capacity (487 ppm and 360 ppm, respectively), which indicates that this property cannot be attributed to a single compound or group of metabolites. The antioxidant capacity of mango peels depends a lot on the phenolic content and the presence of different compounds that could be responsible for the synergy and the variation in its activity [26].

Reactive oxygen species (ROS) are crucial in all wound healing processes. In low concentrations, ROS help regulate signal transmission to eliminate pathogenic microorganisms. However, when these levels rise, oxidative stress is generated, which causes cytotoxicity and damage to the cells surrounding the wound. Therefore, it is important that MEMI chemical compounds have the ability to oxidize ROS [31]. The flavonoids depend on the presence of hydroxyphenolic groups and double bonds, in conjunction with the 4-oxo function of the C ring in its chemical structure, acting on reactive oxygen, mainly superoxide anions, to exert their redox activity; however, it also exerts action on hydroxyl radicals, lipid peroxides, or hydroperoxides. This also blocks its harmful action on cells, such as fibroblasts, keratinocytes, and endothelial cells [32]. Mangiferin also has the ability to stabilize free radicals, reducing oxidative stress in the wound [5].

Acute partial-thickness wounds (superficial wounds) include incisional skin wound patterns and involve immediate primary wound closure after creation. These types of wounds are created using a sharp blade that results in rapid alteration of tissue integrity with minimal collateral damage and can be used to assess the wound healing relationship with a novel product that can aid in lowering the healing time and diminishing scars. Furthermore, this model makes it possible to study the interactions and influences of different cell types, wound contraction, granulation tissue, etc. [10,33].

For this reason, in this study, the incision model was used to evaluate healing efficiency, which includes the observation of wound contraction, tensile strength, and scar formation. Macroscopic observations in the wound contraction experiment distinguished similar characteristics in both groups (MEMI and dexpanthenol): granulation tissue from the third day, drastic reduction in the wound on the seventh day, and definition of the scar on the eleventh. Both groups exceeded 50% closure after the fifth day, so there were no significant differences [34]. We worked with an aqueous extract of *M. indica* leaves in a model of wound healing by excision and mentioned that the contraction of the lesion was gradual and took place in approximately 21 days. In addition, the detachment of the scab was observed on the eighth day in the group treated with the dose of 100 mg/kg/day of the extract, while, in the group without treatment, the contraction took 30 days, and the scab was detached on day 14. It is important to highlight that, although epidermal repair is being evaluated in both models, the mechanism is different since, in the incision model (applied in this study), healing is carried out consisting of the union of edges by means of the epidermal cell proliferation and collagen production. Otherwise, in the excision model, a tissue segment is removed involving the three layers of the skin, carrying out regeneration, which consists of rebuilding the organs and tissues without leaving a scar, for example, after the amputation of a limb [35].

In the histological analysis, tissue lesion sections were identified in mice treated with MEMI, showing an architecture similar to that of normal skin and wound closure greater than that of the dexpanthenol group. However, they did not show significant differences between the MEMI and dexpanthenol groups. This may be due to the presence of phenolic compounds and xanthones since they promote endothelial cell migration. In an in vitro study, the mango extract fractions increased endothelial cell migration in a dose-dependent manner, and the most significant effect is observed with the fraction enriched with phenolic acids and with the one containing phenolic esters and flavonol glycosides. Meanwhile, the effects of isolated compounds (mangiferin and quercetin) induced paradoxical results. On the one hand, quercetin decreased the migration of the same cells and mangiferin increased migration, although it did not increase cell proliferation [5]. This possible mechanism in healing may be due to the multiple bioactive components that may be in the extract, which may synergistically or antagonistically influence the promotion of different molecular responses.

The MEMI tensile strength parameter was significantly different with respect to the dexpanthenol group and the vehicle (surgical gel). Dexpanthenol is an alcoholic analog of pantothenic acid within the family of B complex vitamins (vitamin B5). It is well absorbed through the skin, where it is rapidly converted by enzymatic action into pantothenic acid, a component of the coenzyme A (CoA) important in the metabolism of skin cells. In vitro experiments with human fibroblasts have shown that dexpanthenol helps the proliferation of these cells [36]. Other studies (in vitro and in vivo) have also shown that this drug activates fibroblast proliferation, helps re-epithelialization, and has an anti-inflammatory effect [37,38].

Inflammation is one of the most controversial phases in the healing process since there must be a fine balance so that the response of the immune cells is not blocked or exacerbated, prolonging oxidative stress and deteriorating the proliferative and remodeling phase [31]. That is why the anti-inflammatory activity of MEMI induced by TPA was evaluated. The group treated with the MEMI showed little edema and greater leukocyte cell infiltrate compared to the diclofenac group; thus, both treatments presented significant differences. The epidermal thicknesses of the groups treated with MEMI and diclofenac were lower compared to the group treated only with TPA. These results are similar to another study in which the extract of *M. indica* was evaluated with three different doses (0.5–2 mg per ear) with the model of inflammation induced by arachidonic acid (AA). Nevertheless, compared to our study, they find that there are no significant differences with the reference drug (nimesulide). This possible activity could be attributed to the main compounds of the extract by polyphenols, terpenoids, steroids, fatty acids, and the main compound mangiferin [6].

In addition, it is mentioned that the mechanisms of action of mangiferin aglycone have been studied in in vitro and in vivo models, contributing an inhibition in the expression of COX-2, the production of PGE_2_ [39], LTB4 [6], and the expression of iNOS induced by interleukin (IL)-1β to it. It has also been suggested that mangiferin could be partially responsible for the inhibition of the production of some cytokines, such as TNF-α, IL-4, and IL-5 [40]. This xanthone, together with the phenolic components, is associated with powerful anti-inflammatory effects through the modulation and activation of macrophages.

The main factors that alter the inflammatory phase of the wound-healing process are bacterial infections [8] due to oxidative stress and the lack of care and hygiene in the lesions [41,42,43]. From the moment the epithelium loses its continuity, different microorganisms, such as bacteria, fungi, and antigens, can cause contamination to a critical infection [41]. In particular, wound infections are mainly associated with four bacterial strains: *S. aureus*, *Streptococcus* species, *E. coli*, and *P. aeruginosa* [44,45]. These bacterial strains can produce endotoxins that, in turn, raise the levels of pro-inflammatory cytokines, such as IL-1 and TNF-α. If this continues, the wound can enter a chronic state and fail to heal [46].

In the antibacterial evaluation, the four microorganisms were sensitive to MEMI; despite this, the chloramphenicol group was significantly better with respect to the extract group. Chloramphenicol is a broad-spectrum drug active on most Gram-positive and Gram-negative bacteria, acting at the ribosomal level by inhibiting the protein synthesis of the 50S subunit of the bacterial 70S ribosome. They bind to the transfer ribonucleic acid (RNA) binding site, disrupting protein chain elongation during synthesis [47]. This action has a bacteriostatic effect for most of the pathogens, including those evaluated in this study.

The MIC of MEMI was 2 mg/mL for *S. epidermidis* and 4 mg/mL for *P. aeruginosa*, *E. coli*, and *S. aureus*. These results coincide with those obtained by the agar diffusion method of this same study. Some authors have reported results higher than 2 mg/mL in MIC for *S. aureus* with ethanolic extracts of mango peel [48]. Additionally, with extracts from the bark of the tree of the same fruit, values of 4.88, 3.56, and 3.26 mg/mL were shown against *E. coli*, *S. aureus*, and *P. aeruginosa*, respectively [49]. In the case of chloramphenicol, the MIC values of these last three pathogens were 0.0031, 0.0063, and 0.025 mg/mL; this difference in values may be due to some factors, such as purity, selectivity, as well as the specific mechanism of action of chloramphenicol, which makes it a broad-spectrum antibiotic. However, as with most of these drugs, some strains have shown resistance mechanisms, such as the biosynthesis of enzymes that transfer an acetyl group forming an inactive diacetylated derivative [47].

The antibacterial activity presented by *M. indica* is mainly attributed to its components: phenolic acids, flavonoids, and xanthones, as reported in several investigations [49,50]. It is known that some phenolic compounds (epigallocatechin gallate) and flavonoids (quercetin) can change membrane fluidity and cell morphology, causing the leakage of cytoplasmic material and forming complexes with extracellular and intracellular proteins [51]. Some flavanones have the ability to reduce the detection of quorum sensing (QS) in *P. aeruginosa* (strain PAO1) [52] in such a way that they disrupt biofilm networks. Mangiferin has also been reported to have iron-chelating activity [53], an essential element for bacterial proliferation, having its active site with enzymes that participate in DNA synthesis. Therefore, the presence of phenolic acids, flavonoids, and xanthones in crude extracts could potentiate antibacterial activity.

Certain that MEMI is a product that meets several healing properties, it was important to know its safety via topical application. When evaluating the toxicity of MEMI, it did not show any harmful signs during the 14 days of experimentation with the maximum doses (2000 mg/Kg). According to the bibliography, no antecedents were found about the acute toxicity of mango peel extracts. Although, Maisuthisakul and Gordon in 2009 [54] determined the acute skin irritation (OECD 404) using 0.5 g of the ethanolic extract of mango seeds, placing it on patches that, in turn, were adhered to the shaved skin of rabbits and were monitored in different times. Similarly, the aqueous extract of the mango tree bark was evaluated with protocols 423 (2000) and 434 (2004) of the OECD, and there were no signs of toxicity, deaths, or histological alterations observed in the necropsy of the animals in any study [55].

## 5. Conclusions

The incisional wound model is widely used and accepted in the scientific community because it has several advantages: it allows evaluating the closure of the lesion through the rapid and efficient union of the injured edges and the formation of granulation tissue and new epithelium. The biomechanics of healing can be easily identified, and the aesthetics of scars elucidated. Additionally, the standardization is simple, and the cost is low even with a variable sample size. This model was applied in the evaluation of the healing effect of MEMI, suggesting that phenolic compounds, mainly flavonoids, and mangiferin most likely act as antibacterial and anti-inflammatory agents, regulate oxidative stress, and provide resistance to traction and inhibit or activate enzymes for the production and maturation of collagen. Therefore, this study demonstrates the multiple targets that MEMI can present during the healing process, including some local factors that can inhibit it, as well as promote a possible use of the by-product of this fruit.

## Figures and Tables

**Table 1:** HPLC-DAD and HPLC-MS analysis of the standard glycosylated-mangiferin and the mangiferin detected in the extract of *M. indica*.

Name	Retention Time (min)		λ_max_ (nm)	Parent ion (*m*/*z*) [M-H]^−^	Relative Error (ppm)
	HPLC-DAD	HPLC-MS			
Mangiferin (standard)	9.373	2.019	258, 318, 366	421.0462	−4.8
Mangiferin (extract)	9.277	1.862	258, 318, 366	421.0530	5.05

**Table 2:** Total content of Phenols, flavonoids and antioxidant capacity of *Mangifera indica* methanolic extract.

TPC (GAE/g)	136 mg
TFC (QE/g)	101.66 mg
SA_50_	36.33 μg/mL

TPC: total phenol content; TFC: total flavonoid content; (GAE)/g = mg: gallic acid equivalent per gram of dried extract; (QE)/g = mg of quercetin equivalent per gram of dried extract.

**Table 3:** Antibacterial activity of MEMI (inhibition diameter in mm).

	Mean ± Standard Deviation			
Bacteria	Positive Control (Chloramphenicol 25 µg)	Inhibition Halos (mm)	MIC (mg/mL)	MBC (mg/mL)
*S. aureus*	20.4 ± 0.5	9.8 ± 0.4 ^a^	4.0	8.0
*S. epidermidis*	21.8 ± 0.4	13.8 ± 1.9 ^a^	2.0	8.0
*P. aeruginosa*	13.2 ± 0.4	11.6 ± 0.5 ^a^	4.0	8.0
*E. coli*	20.8 ± 0.4	10.6 ± 0.4 ^a^	4.0	8.0

Effect of extract methanolic of *Mangifera indica* (MEMI) on bacteria strains. *S. epidermidis* was the most sensitive. The negative control (10 μL of MeOH) had no effect on the strains; therefore, the data are not shown. ^a^ There are significant differences with respect to the control group (*p* < 0.05). MIC: Minimum inhibitory concentration, MBC: Minimum bactericide concentration.

**Table 4:** Observations of changes in the animal welfare parameters in females threatened with 2000 mg/Kg MEMI.

Observations	30 min			2 h			4 h			24 h			48 h			72 h			7 Days			14 Days		
	C	V	M	C	V	M	C	V	M	C	V	M	C	V	M	C	V	M	C	V	M	C	V	M
Skin irritation	N	N	2 × 1n	N	N	1 × 2n	N	N	N	N	N	N	N	N	N	N	N	N	N	N	N	N	N	N
Piloerection	N	N	2 × 1n	N	N	1 × 2n	N	N	N	N	N	N	N	N	N	N	N	N	N	N	N	N	N	N
Eyes	N	N	N	N	N	N	N	N	N	N	N	N	N	N	N	N	N	N	N	N	N	N	N	N
Mucous membranes	N	N	N	N	N	N	N	N	N	N	N	N	N	N	N	N	N	N	N	N	N	N	N	N
Irritation	N	N	N	N	N	N	N	N	N	N	N	N	N	N	N	N	N	N	N	N	N	N	N	N
Brething	N	N	N	N	N	N	N	N	N	N	N	N	N	N	N	N	N	N	N	N	N	N	N	N
Tremors	N	N	N	N	N	N	N	N	N	N	N	N	N	N	N	N	N	N	N	N	N	N	N	N
Convulsions	N	N	N	N	N	N	N	N	N	N	N	N	N	N	N	N	N	N	N	N	N	N	N	N
Salivation	N	N	N	N	N	N	N	N	N	N	N	N	N	N	N	N	N	N	N	N	N	N	N	N
Diarrhea	N	N	N	N	N	N	N	N	N	N	N	N	N	N	N	N	N	N	N	N	N	N	N	N
Lethargy	N	N	1 × 2n	N	N	N	N	N	N	N	N	N	N	N	N	N	N	N	N	N	N	N	N	N
Sleep	N	N	N	N	N	N	N	N	2 × 1n	N	N	N	N	N	N	N	N	N	N	N	N	N	N	N
Coma	N	N	N	N	N	N	N	N	N	N	N	N	N	N	N	N	N	N	N	N	N	N	N	N
Mortality	N	N	N	N	N	N	N	N	N	N	N	N	N	N	N	N	N	N	N	N	N	N	N	N

C = Control; V = Vehicle; M = Methanolic extract of *Mangifera indica*; N = Normal; 1 × 2n = 1 affected, 2 normal; 2 × 1n = 2 affected, 1 normal.

## Data Availability

The data used to support the findings of this study are available from the corresponding author upon request.

## Sample Availability

Samples of the compounds are not available from the authors.
